# A multiplexed light-matter interface for fibre-based quantum networks

**DOI:** 10.1038/ncomms11202

**Published:** 2016-04-05

**Authors:** Erhan Saglamyurek, Marcelli Grimau Puigibert, Qiang Zhou, Lambert Giner, Francesco Marsili, Varun B. Verma, Sae Woo Nam, Lee Oesterling, David Nippa, Daniel Oblak, Wolfgang Tittel

**Affiliations:** 1 Institute for Quantum Science and Technology, University of Calgary, 2500 University Drive NW, Calgary, Alberta, Canada T2N 1N4; 2 Department of Physics and Astronomy, University of Calgary, 2500 University Drive NW, Calgary, Alberta, Canada T2N 1N4; 3 Applied Physics Division, Jet Propulsion Laboratory, California Institute of Technology, 4800 Oak Grove Drive, Pasadena, California 91109, USA; 4 National Institute of Standards and Technology, Boulder, Colorado 80305, USA; 5 Battelle, 505 King Avenue, Columbus, Ohio 43201, USA; 6 Present address: Department of Physics, University of Ottawa, 150 Louis Pasteur, Ottawa, Ontario, Canada K1N 6N5

## Abstract

Processing and distributing quantum information using photons through fibre-optic or free-space links are essential for building future quantum networks. The scalability needed for such networks can be achieved by employing photonic quantum states that are multiplexed into time and/or frequency, and light-matter interfaces that are able to store and process such states with large time-bandwidth product and multimode capacities. Despite important progress in developing such devices, the demonstration of these capabilities using non-classical light remains challenging. Here, employing the atomic frequency comb quantum memory protocol in a cryogenically cooled erbium-doped optical fibre, we report the quantum storage of heralded single photons at a telecom-wavelength (1.53 μm) with a time-bandwidth product approaching 800. Furthermore, we demonstrate frequency-multimode storage and memory-based spectral-temporal photon manipulation. Notably, our demonstrations rely on fully integrated quantum technologies operating at telecommunication wavelengths. With improved storage efficiency, our light-matter interface may become a useful tool in future quantum networks.

## Abstract (web summary)

Scalable networks for processing and distribution of quantum information using photons can be achieved by using multiplexed quantum states. Here, the authors report frequency-multimode storage and spectral-temporal photon manipulation of heralded single photons at telecom wavelength, in a fully integrated setting.

## Graphical abstract

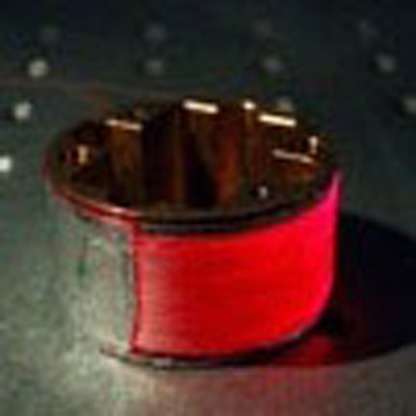

Multiplexing, particularly in the form of wavelength division multiplexing, is key for achieving high data rates in modern fibre-optic communication networks. The realization of scalable quantum information processing demands adapting this concept, if possible using components that are compatible with the existing telecom infrastructure. One of the challenges to achieve this goal is to develop integrated light-matter interfaces that allow storing and processing multiplexed photonic quantum information. In addition to efficient operation, ease of integration and feed-forward controlled recall, the suitability of such interfaces depends on the storage time for a given acceptance bandwidth, that is, the interface's time-bandwidth product, as well as its multimode storage and processing capacities. It is important to note that although the multimode capacity of a light-matter interface determines the maximum number of simultaneously storable and processable photonic modes, the time-bandwidth product only sets an upper bound to the multimode capacity for temporally and/or spectrally multiplexed photons.

Over the past decade there has been significant progress towards the creation of such light-matter interfaces. One promising approach is based on a far off-resonant Raman transfer in warm atomic vapour, for which a time-bandwidth product of 5,000 has been reported^1^. However, owing to noise arising from (undesired) four-wave mixing, the storage and recall of quantum states of light have proven elusive^2^. This problem can be alleviated by implementing the Raman protocol in other media, for example, diamonds (with storage in optical phonon modes) and laser-cooled atomic ensembles, for which time-bandwidth products around 22 have been obtained with non-classical light^3^,^4^. However, the multimode operation of any Raman-type memory and hence the utilization of the potentially achievable large time-bandwidth products will remain challenging because of unfavourable scaling of this scheme's multimode capacity with respect to optical depth^5^.

Another promising avenue for a multiplexed light-matter interface is the atomic frequency comb (AFC)-based quantum memory scheme in cryogenically cooled rare-earth ion-doped materials^6^,^7^. An attractive feature of this approach is that, unlike in other protocols, the multimode storage capacity is independent of optical depth; it is solely given by the time-bandwidth product of the storage medium, which can easily go up to several thousands because of the generally large inhomogeneous broadening (enabling large storage bandwidth) and narrow homogeneous linewidth (allowing long storage times) of optical transitions in rare-earth ion-doped materials. This aspect has already allowed several important demonstrations, including the simultaneous storage of 64 and 1,060 temporal modes by AFCs featuring pre-programmed delays^8^,^9^, 5 temporal modes by an AFC with recall on-demand^10^ and 26 spectral modes supplemented with frequency-selective recall^11^, respectively. Despite the importance of these demonstrations, they were restricted to the use of strong or attenuated laser pulses rather than non-classical light, as required in future quantum networks. The only exception is the very recent demonstration of the storage of photons, emitted by a quantum dot, in up to 100 temporal modes^12^.

In this paper, we present a spectrally multiplexed light-matter quantum interface for non-classical light. More precisely, we demonstrate large time-bandwidth product and multimode storage of heralded single photons at telecom wavelength by implementing the AFC protocol in an ensemble of erbium ions. As an important feature for future quantum networks, our demonstrations rely on fully integrated quantum technologies, that is, a fibre-pigtailed LiNbO_3_ waveguide for the generation of heralded single photons by means of parametric down-conversion, a commercially available, cryogenically-cooled erbium-doped single-mode fibre for photon storage and manipulation, and superconducting nanowire devices for high-efficiency single-photon detection.

## Results

### Experimental setup

**Figure 1:**
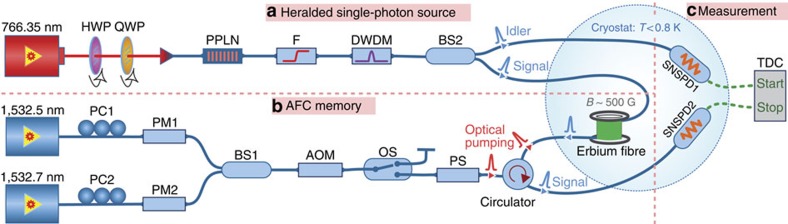
Experimental setup. (**a**) Heralded single-photon source. Narrow linewidth continuous-wave (CW) light at 766.35 nm wavelength and with 100 μW power is sent to a fibre-pigtailed, periodically poled lithium niobate (PPLN) waveguide that is heated to 52.8 °C. A quarter-wave-plate (QWP) and a half-wave-plate (HWP) match the polarization of the light to the crystal's C axis to maximize the nonlinear interaction. Spontaneous parametric down conversion (SPDC) in the PPLN crystal results in frequency degenerate photon pairs with 40 nm bandwidth, centred at 1,532 nm wavelength. The residual pump light at 766.35 nm is suppressed by 50 dB by a filter (F) and the bandwidth of the created photons is filtered down to 50 GHz using a dense-wavelength-division-multiplexer (DWDM). The filtered photon pairs are probabilistically split using a beam-splitter (BS2). The detection of one member (the ‘idler' photon) heralds the presence of the other (the ‘signal' photon), which is directed to the input of the AFC memory. (**b**) Quantum memory. The quantum memory is based on an erbium-doped fibre that is exposed to a magnetic field of 600 G and cooled to a temperature below 1 K. Light from two independent CW lasers with wavelengths of 1,532.5 and 1,532.7 nm, respectively, is used to spectrally tailor the inhomogeneously broadened 1,532 nm absorption line of erbium through frequency-selective optical pumping into one or several atomic frequency combs. Towards this end, phase-modulators (PM1 and PM2) followed by an acousto-optic modulator are used to generate chirped pulses with the required frequency spectrum. The optical pumping light from the two lasers is merged on a beam-splitter (BS1) and enters the erbium fibre from the back via an optical circulator. Polarization controllers (PM1 and PM2) match the polarization to the phase-modulators' active axes, and the polarization scrambler ensures uniform optical pumping of all erbium ions in the fibre^13^. (**c**) Measurement unit. The detection of the heralding photon (‘idler') and subsequently the ‘signal' photon is performed by two superconducting nano-wire single-photon detectors (SNSPD1 and SNSPD2) maintained at the same temperature as the memory. The coincidence analysis of the detection events is performed by a time-to-digital converter (TDC).

Our experimental setup, illustrated in Fig. 1, is composed of an integrated, heralded single-photon source, an AFC-based erbium-doped fibre memory and a measurement unit including two superconducting nanowire single-photon detectors (SNSPDs).

We generate pairs of energy-time quantum correlated telecom-wavelength photons—commonly referred-to as ‘signal' (‘s') and ‘idler' (‘i')—by sending pump light from a continuous-wave laser operating at 766 nm wavelength to a periodically poled lithium niobate (PPLN) waveguide, as shown in Fig. 1a. Spontaneous parametric down conversion (SPDC) based on type-0 phase matching results in the creation of frequency-degenerate photon pairs centred at 1,532 nm wavelength and having a bandwidth of ∼40 nm. We note that the PPLN waveguide is fibre pigtailed at both input and output faces (that is, for the pump light as well as the down-converted photons), which makes the source alignment free. After filtering away the remaining pump light, the spectra of the generated photons are filtered down to 50 GHz resulting in a photon-pair generation rate of 0.35 MHz. The ensuing photons are probabilistically separated into two standard telecommunication fibres using a 50/50 fibre-optic beam splitter (BS2). One member of each split pair is sent into an SNSPD featuring a system detection efficiency of around 70% (see the Methods for details of the SNSPDs). Its electronic output heralds the other member, which travels through standard telecommunication fibre to our light-matter interface (for more details about the heralded single-photon source see the Supplementary Note 1).

**Figure 2:**
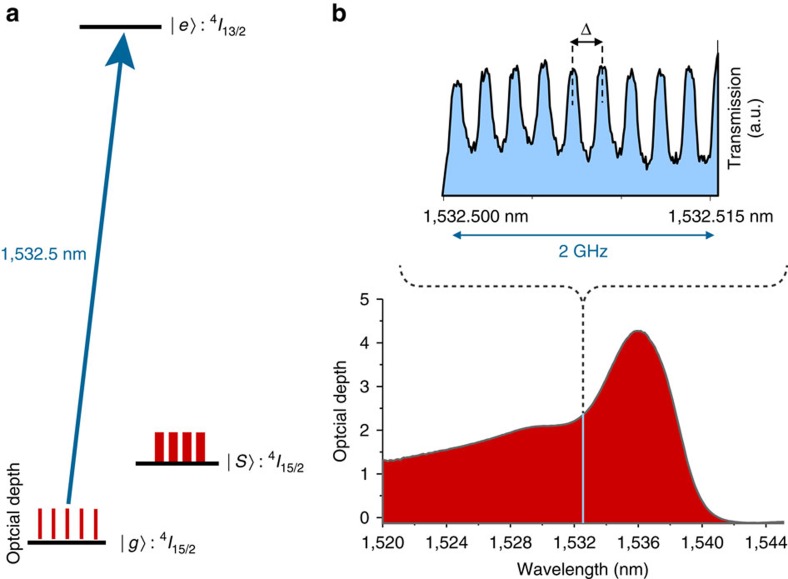
Quantum memory. (**a**) Simplified level scheme of Er^3+^ in silica glass. Frequency-selective optical pumping from the ^4^*I*_15/2_ electronic ground state (|g〉) via the ^4^*I*_13/2_ excited state (|e〉) into an auxiliary (spin) state (|s〉) allows spectral tailoring. (**b**) Inhomogeneous broadening and AFC structure. The inhomogeneously broadened optical absorption line of erbium ions in silica fibre at 1 K extends roughly from 1,500 to 1,540 nm wavelength. A 2-GHz-wide section of a 16-GHz-wide comb at 1,532.5 nm with teeth spacing Δ=200 MHz is shown.

To store or manipulate the heralded single photons, we prepare an AFC-based memory in a 20-m-long erbium-doped silica fibre maintained at a temperature of 0.6–0.8 K and exposed to a magnetic field of 600 G (see Fig. 1b and the Methods for details). The memory relies on spectral tailoring of the inhomogenously broadened, ^4^*I*_15/2_↔^4^*I*_13/2_ transition in erbium into a comb-shaped absorption feature characterized by the teeth spacing, Δ, as shown in Fig. 2a. The spectral tailoring is performed by frequency-selective optical pumping of ions into long-lived auxiliary (spin) levels. When an input photon is absorbed by the ions constituting the comb, a collective atomic excitation is created. It is described by:


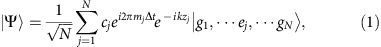


where *N* is the total number of addressed atoms, *k* is the optical wave number and 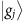 and 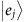 are the *j*'th atom's ground and excited states, respectively. The detuning of the atom's transition frequency from the photon carrier frequency is given by *m*_*j*_Δ, whereas *z*_*j*_ is the position of the atom measured along the propagation direction of the light, and the factor *c*_*j*_ depends on both the resonance frequency and position of the atom. Owing to the periodic nature of the AFC, the atomic excitation is converted back to photonic form and the input photon is re-emitted in the originally encoded state after a storage time given by the inverse of the peak spacing, *t*_storage_=1/Δ, as shown in Fig. 2b. The use of erbium-doped fibre is particularly attractive for AFC-based quantum memory because of its polarization insensitive operation at wavelengths within the telecom C-band^13^, its ability to store photonic entanglement in combination of ease of integration with standard fibre infrastructure^14^, and its large usable inhomogeneously broadened absorption line^15^, which allows for multimode storage and manipulation of photons with large bandwidth, as detailed below.

Finally, we detect the heralded photons after storage/manipulation using a second SNSPD, featuring similar performance as that used to detect the heralding photon. All detection signals are sent to a time-to-digital converter (TDC) to perform time-resolved coincidence measurements. This allows us to calculate the cross-correlation function


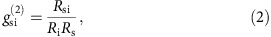


where *R*_si_ is the rate of coincidence detections, and *R*_s_ and *R*_i_ are the single detection count-rate for ‘signal' and ‘idler' photons, respectively. A classical field satisfies the Cauchy–Schwarz inequality 
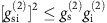
 where 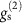 and 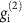 are second-order autocorrelation functions for ‘signal' and ‘idler' modes. For photons derived from an SPDC process, the second-order autocorrelation is bounded by 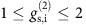 (ref. 16). Consequently, measuring a cross-correlation 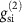
>2 violates the inequality and thus verifies the presence of quantum correlations between the members of the photon pairs. We characterize our source for different SPDC pump powers (shown in Supplementary Fig. 1), finding that the cross-correlation function exceeds 1,000 for all powers. As 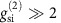 implies that the heralded autocorrelation function of the stored signal photons is 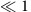 (ref. 17), we denote these as single photons. The details of how 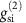 is obtained from the coincidence counts are given in the Methods.

### Measurements

**Figure 3:**
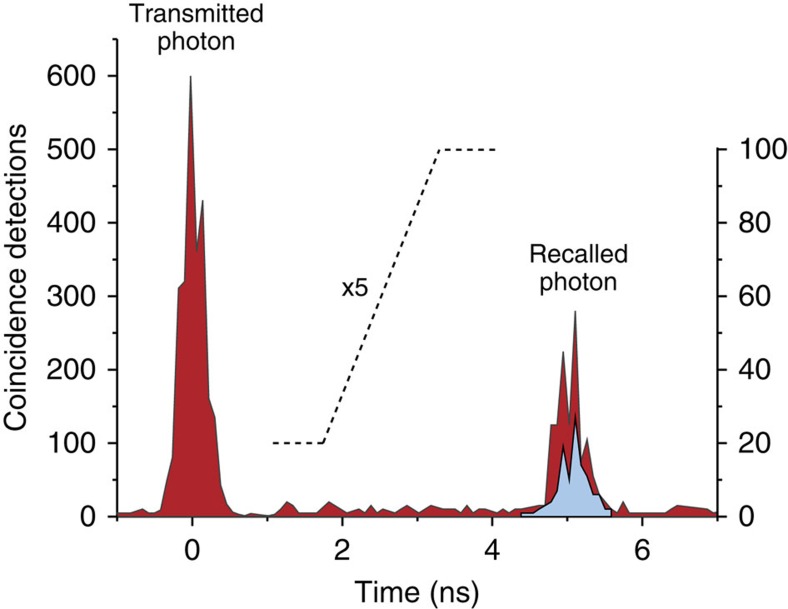
Reversible mapping of broadband and heralded single photons. Heralded telecom-wavelength photons, centred at 1,532.7 nm wavelength and having a bandwidth of 50 GHz, are mapped onto the AFC memory and recalled after time 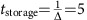 ns. The histogram shows time-resolved coincidence detections collected over 3 min. Owing to non-unit absorption probability by the AFC as well as bandwidth mismatch between the spectra of the photons and the AFC, a significant portion of the input photons is directly transmitted and detected at zero delay. The light blue highlighted section bounded by a dotted line in the trace of the recalled photon (counts scaled by a factor of five) corresponds to the measurement in which the total AFC bandwidth was 8 GHz; the red section corresponds to a measurement using a 16-GHz-wide AFC.

First, we investigate the storage of broadband heralded single photons in an AFC memory—prepared using a single optical pumping laser at 1,532.5 nm—with a total bandwidth of 8 GHz and 200 MHz tooth spacing (corresponding to 5 ns storage time), as shown in Fig. 2b. The recalled photons are shown as a light-blue trace in the histogram of coincidence detections in Fig. 3. Analysing the correlations between signal and recalled idler photons, we find 
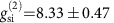
, which shows that the heralded photons after—and hence also before—storage, are indeed non-classical, and thus confirms the quantum nature of our light-matter interface. To improve the storage efficiency and thus the signal-to-noise ratio in the coincidence counts, we increase the storage bandwidth of the memory to 16 GHz—recall that the bandwidth of the input photons is 50 GHz. Owing to restrictions imposed by the level structure of erbium (see Supplementary Fig. 2 for details), the bandwidth increase is achieved by generating an additional 8-GHz-wide section separated from the first by around 20 GHz from edge to edge. This entails using two independent lasers operating at 1,532.5 and 1,532.7 nm wavelength for preparation of the two spectrally separated AFCs. As expected, this leads to an improvement of the overall memory efficiency and thus coincidence count rate from 0.59±0.06 to 1.29±0.13 Hz (see Fig. 3 for details), and an increase of 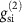 to 18.2±0.9. The number of AFCs, and thus the total storage bandwidth, can be further increased by employing additional pump lasers.

**Figure 4:**
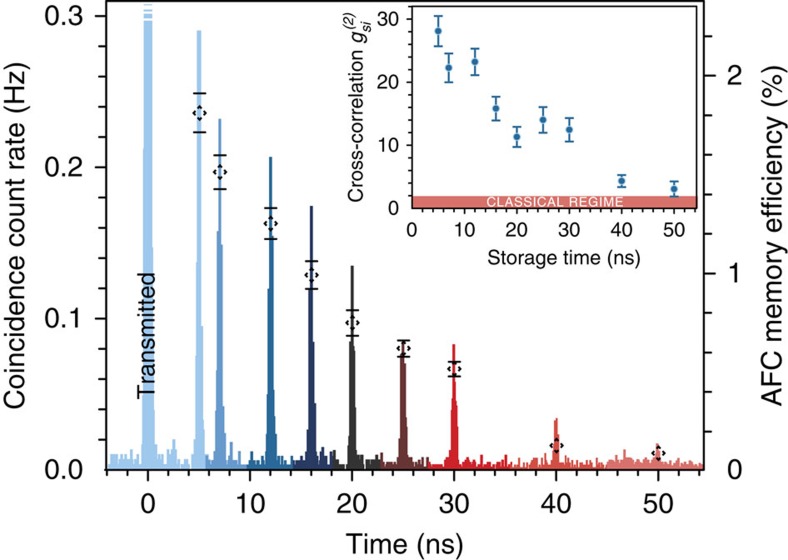
Quantum storage with large time-bandwidth product. 16-GHz-wide AFC regions with teeth spacing ranging from 200 to 20 MHz are subsequently programmed to store heralded single photons for 5–50 ns. The histograms show measured coincidence rates for recalled photons for each storage time, and the dashed diamonds depict the corresponding memory efficiencies (see Supplementary Note 3 for details). Note that in this figure and henceforth the transmitted pulse at *t*=0 exceeds the vertical scale and thus is capped at the top. Experimentally obtained 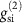 for each storage time are shown in the inset. The measurement time varied between 5 and 15 min. All error bars are standard deviations derived from Poissonian counting statistics.

Second, to demonstrate quantum storage with large time-bandwidth product, we extend the memory storage time from 5 to 50 ns. To this end, we programme a 16-GHz-wide spectral region with two AFCs having tooth spacing ranging from 200 to 20 MHz. For each case, we map heralded photons onto the double AFC and collect coincidence statistics for the recalled photons as shown in Fig. 4. As further detailed in the Supplementary Information of ref. 14, decoherence effects and imperfect preparation of the AFCs decreases the memory efficiency as the storage time increases. Nevertheless, as shown in the inset of Fig. 4, 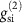 still remains above 2 up to the maximal storage time of 50 ns, and thus we demonstrate the storage of non-classical light with a time-bandwidth product up to 800 (that is, 16 GHz × 50 ns). This is an improvement by close to a factor of 40 over that obtained in Raman-based memories^4^ and a factor of 3 over the recent AFC-based memory demonstration^12^, respectively. We emphasize that this comparison of time-bandwidth products does not reflect the difference in multimode storage capacities of the Raman and AFC-based implementations.

**Figure 5:**
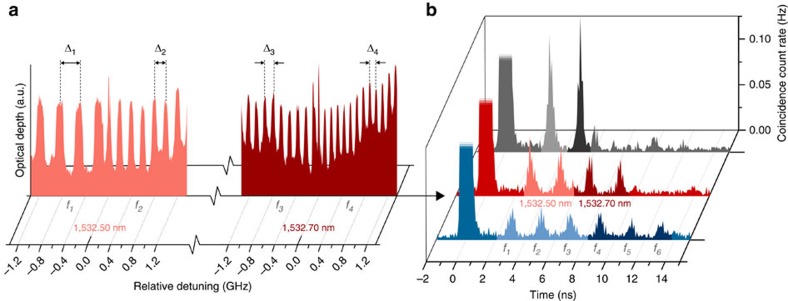
Multimode quantum storage. (**a**) Creation of AFCs. The total, currently addressable bandwidth of 18 GHz is divided into different spectral sections, each featuring an AFC with distinct peak spacing. Depicted is the case of four, 4.5-GHz-wide AFCs, created using two lasers operating at 1,532.50 and 1,532.70 nm. The AFCs feature peak spacings of 333, 200, 143 and 111 MHz, corresponding to storage times of 3, 5, 7 and 9 ns, respectively. For each AFC, only a 1.3-GHz-wide section is shown. (**b**) Storage and recall. When broadband heralded photons with 50 GHz bandwidth are mapped onto a two-section AFC, where each section extends over 9 GHz bandwidth and has a peak spacing of 333 and 200 MHz, respectively, they are stored in two spectral modes and retrieved in two spectro-temporal modes, as shown in the back trace. Decreasing the bandwidth per AFC allows increasing the number of AFCs (spectral modes), as demonstrated with the storage of four and six spectral modes in the middle and the front trace, respectively. The modes are labelled *f*_*i*_. For each mode, 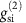 is measured to be larger than 2 (see Table 1).

Third, to establish the multimode operation of our quantum light-matter interface, we divide the total currently accessible bandwidth—18 GHz for this experiment—into different numbers of AFC sections, and programme each section with a different storage time ranging from 3 to 13 ns. This allows identifying photons stored in different frequency modes (that is, different AFC sections) through their recall time. In succession, we perform measurements with: two AFCs of 9 GHz bandwidth; then four AFCs of 4.5 GHz bandwidth (this case is shown in Fig. 5a); and finally six AFCs of 3 GHz bandwidth. The corresponding histograms of coincidence detections, depicted in Fig. 5b, confirm that two, four and six modes, respectively, have been stored simultaneously. For each recalled spectro-temporal mode, we find that 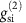, listed in Table 1, exceed the classical limit of two, thereby confirming multimode storage of non-classical states of light in matter.

**Figure 6:**
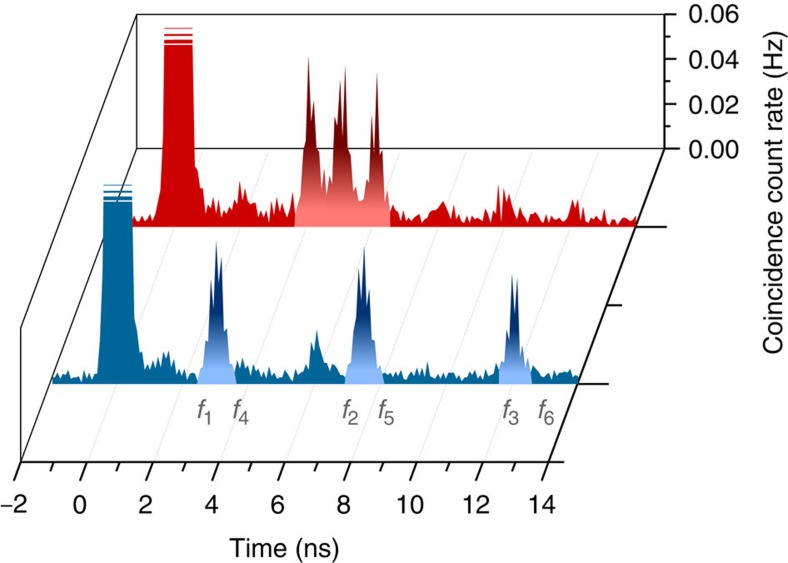
Pulse manipulation. The AFC memory with total bandwidth of 2 × 8 GHz=16 GHz is tailored such that simultaneously absorbed photons in six spectral modes (from *f*_1_ to *f*_6_), each of 2.65 GHz bandwidth, are recalled in three temporal modes spaced either by 4.5 ns (front trace), or by 1 ns (back trace).

The frequency-to-time mapping described above was used to unambiguously distinguish between different frequency modes. This is convenient in our proof-of-principle demonstration, but may not be required in all applications. However, the ability to flexibly reconfigure our light-matter interface in terms of the number, bandwidth, storage time and frequency separation of the AFCs lends itself to versatile manipulation at the quantum level. In particular, it allows modifying the temporal shape of single photons by recalling different spectral modes at the same time and with adjustable phases. As an illustration, Fig. 6 shows two different mappings of six spectral modes onto three distinct temporal modes. We note that additional processing, for example, pulse sequencing and temporal compressing, relying on the frequency-to-time mapping is possible by combining AFCs with frequency shifters, as demonstrated in ref. 18.

## Discussion

A central feature of our demonstration is the large multiplexed storage and manipulation capacity of our atomic interface for non-classical light. Yet, as noted above, there are clear avenues to increase this even further. Our additional investigations depicted in Supplementary Fig. 3 show that at least a 14-nm span of the erbium absorption line is suitable for quantum state storage. If we generate 8 GHz broad AFCs separated by 20 GHz, this yields a total of 64 AFCs covering a combined bandwidth of 64 × 8 GHz=512 GHz. Assuming 50 ns storage time, one could thus reach a time-bandwidth product of more than 512 GHz × 50 ns≈25,000, which is equal to the number of temporal—spectral modes that could be stored simultaneously.

On the other hand, a number of improvements are required for our multiplexed light-matter interface to be truly suitable for use in future quantum networks. First, imperfect optical pumping during the preparation of the AFCs currently results in a storage efficiency at 0.6 K of 1–2% (see the Supplementary Information of ref. 14). However, as we described in ref. 15 and in Supplementary Note 2, we expect that it is possible to substantially improve the optical pumping, for example, with lower temperatures and smaller erbium concentrations, and hence to achieve a significantly higher efficiency under certain conditions.

Second, the ∼10-ms radiative lifetime of the ^4^*I*_15/2_↔^4^*I*_13/2_ transition in erbium fundamentally limits the coherence time to 20 ms. However, coupling to so-called two-level systems^19^, which are intrinsic to disordered materials such as glass fibres, reduces the coherence time, and the attainable storage time is hence currently restricted to a few tens of nanoseconds. Although we anticipate that this value increases with smaller doping concentration and lower temperature^20^,^21^ (see Supplementary Information of ref. 14 for further discussions), it is an open question whether or not it is possible to extend storage times to hundreds of microseconds, which would, for example, allow building a quantum repeater based on spectral multiplexing^11^. Yet, we note that not all applications of quantum memory require long storage times, and that the figure of merit for multiplexing schemes is the time-bandwidth product. Hence, our light-matter interface can be useful even without long storage times.

Finally, we point out that our storage device provides pre-programmed delays, which is sufficient for increasing the efficiency of multi-photon applications if one incorporates external elements that allow feed-forward-based mode-mapping, for example, frequency shifters^11^. However, for some applications, it may be desirable to perform the mode mapping during storage—a familiar example being storage combined with read-out on demand, that is, the possibility to select the recall time after the photon has been stored. One option to enable this feature is to utilize the Stark effect, which allows ‘smearing' out the AFC peaks—whose spacing determines the recall time—after photon absorption^22^. This would inhibit the rephasing of the collective excitation and hence reemission of light after *t*_storage_=1/Δ, and lead to the possibility to recover the original photon a time *t*_storage_=*n*/Δ after absorption, where *n* is an integer. Another possibility is to map the optical coherence—created by the photon absorption—onto a long-lived spin level (spin-wave storage)^23^,^24^, for example, a hyperfine level (caused by the non-zero nuclear spin of ^167^Er^3+^)^25^,^26^ or a superhyperfine level (caused by the interaction of Er with co-dopants in the host)^27^. However, this possibility remains challenging because of the complex and not fully characterized structure of these levels in erbium-doped fibres. Moreover, this approach would limit the memory bandwidth appreciably because of the intrinsically small splitting of nuclear spin levels. But provided these levels feature long coherence times, and thus storage duration, the large time-bandwidth product needs not be affected significantly.

Provided that the performance of our light-matter interface is improved as discussed, it can advance several quantum photonic applications. For instance, by programming AFCs with different storage times into different spectral regions, it can serve as a time-of-flight spectrometer, which would, for example, allow fine-grained, spectrally resolved photon measurements, including two-photon Bell-state measurements in a quantum repeater architecture based on frequency multiplexing^11^. As a second example, our interface can be used to spectrally and temporally tailor single photon wave packets, as exemplified in our demonstrations, and thereby adapt their properties for subsequent interfacing with other quantum devices. More generally, by combining the interface with feed-forward-controlled frequency shifters, for example, broadband electro-optic phase modulators, it can be turned into a programmable atomic processor for arbitrary manipulation of photonic quantum states encoded into time and/or frequency^18^. This would find application in optical quantum computing in a single-spatial mode^28^ or in atomic-interface-based photonic quantum state processing^29^,^30^,^31^,^32^. In addition, we note that the large multiplexing capacity of our light-matter can be increased further by adapting multiplexing in spatial degree^33^, and/or angular orbital momentum degree^34^ with use of multimode erbium-doped fibres, as envisioned for future fibre-based classical and quantum networks^35^.

In conclusion, we have presented a quantum light-matter interface that is suitable for multiplexed quantum-information-processing applications. More precisely, we have demonstrated a large time-bandwidth-product memory capable of multimode storage of non-classical light created via spontaneous parametric down-conversion, and showed that our light-matter interface can be employed for quantum manipulation of broadband heralded single photons. The fully pigtailed photon source and the fibre-based memory, along with telecommunication-wavelength operation and commercial availability, make our demonstration important in view of building future fibre-based quantum networks.

## Methods

### Erbium doped silica fibre

We use a 20-m long, commercially available single-mode, 190 p.p.m.-wt erbium-doped silica fibre specified to have 0.6 dB m^−1^ absorption at 1,532 nm wavelength at room temperature. At 1 K, we measure 0.1 dB m^−1^ absorption at 1,532 nm wavelength (see Fig. 4 for details). In addition to Er, the fibre is co-doped with P, Al and Ge. The fibre is spooled in layers around a copper cylinder with ∼4 cm diameter that is thermally contacted with the base plate of an adiabatic demagnetization refrigerator maintained at about 0.6–0.8 K and exposed to a field of ∼600 G inside a superconducting solenoid magnet. This setup induces ∼70% bending loss from input to output, mainly because of the insufficiently large diameter of the erbium-fibre spool. The fibre is fusion-spliced to standard single-mode fibres (SMF-28) at each end with less than 5% loss per splice.

### Preparation of AFC

The memory is prepared using the setup described in Fig. 1. Frequency chirped laser pulses, applied 500 times per pumping cycle of 500 ms duration, allow frequency-selective optical pumping of erbium ions into a long-lived Zeeman level, and hence the formation of peaks and troughs of the AFC. A polarization scrambler randomly changes the polarization of the pump light every 500 μs to ensure that heralded photons, which propagate counter to the optical pumping light, are absorbed regardless their polarization state^13^. Upon completion of the optical pumping, a significant portion of the erbium ions accumulate in the auxiliary ground-state Zeeman-level |*s*〉 (see Fig. 2 for details), whose decay at 0.7 K and under a magnetic field of 600 G is characterized by two lifetimes of 1.3 and 26 s (ref. 15). However, a considerable amount of atoms remains in excited level |*e*〉, and subsequent spontaneous emission from these would mask all recalled photons. Thus, to eliminate this spontaneous emission noise, we set a wait time of 300 ms, which is significantly larger than the 11-ms exited level lifetime. Following the wait time, we store and retrieve many heralded single photons during a 700-ms measurement time. The retrieval efficiency of our AFC is about 1–2%, which is primarily limited by residual absorption background as a result of incomplete population transfer during the optical pumping, (see Supplementary Information of ref. 14 for further details)

### Superconducting nanowire single-photon detectors

The detection of the telecom-wavelength photons is carried out by a set of SNSPDs^36^ attached to the base plate of the adiabatic demagnetization refrigerator and maintained at the same temperature as the memory. In our setup, the tungsten silicide (WSi) SNSPDs have efficiencies of ∼70%, which includes the loss due to fibre-splices and bends. Their detection efficiencies exhibit a small polarization dependence of ∼5%. The time jitter of the detectors is around 250 ps, which allows us to resolve detection events separated by 1 ns (see Fig. 6 for details). We measure the dark count rate of the detectors to be ∼10 Hz, which results in a negligibly small contribution to accidental coincidences and hence high signal-to-noise ratios for the coincidence detections of the recalled photons.

### Coincidence and *g* ^(2)^ measurements

All detection signals are directed to a TDC, which allows recording detection times with a resolution of 80 ps. The detection of the heralding (‘idler') photon on SNSPD1 is used to trigger (start) the TDC. The other member of the pair (the ‘signal' photon) is detected by SNSPD2—either as recalled (delayed) photon after storage, or as a directly transmitted photon through the erbium fibre that features no extra delay—and sent to the TDC, which records the time interval from the trigger signal. This allows us to generate histograms of the time-resolved coincidence detections between ‘signal' and ‘idler' photons (see, for example, Fig. 3).

To calculate 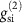 using equation 2, we extract the values for the rates of the coincidence detection *R*_si_(*t*=0) and the individual detections *R*_s_ and *R*_i_ from coincidence histograms. Here we have explicitly stated the time difference between the coincidence detections to be 0. We note that, as the photon-pair generation process is spontaneous, there is no statistical correlation between subsequent pair generation events. Hence we can re-write the product *R*_s_*R*_i_ as *R*_si_(*t*≠0), that is, as the coincidence detection rates for ‘signal' and ‘idler' photons that are not members of the same photon pair, which is often referred to as accidental coincidence count-rate^37^. We extract *R*_si_(*t*=0) from a coincidence histogram by counting all detections within the ‘coincidence peak' that is centred at time *t*_0_ and has width *t*_p_, and normalizing this number by measurement time and coincidence window width *t*_p_. Similarly, *R*_si_(*t*≠0) is evaluated by appropriate normalization of coincidence counts taken in a window of width *t*_bg_ that is adjacent to the ‘coincidence peak'. Hence, to evaluate 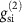 from experimental histograms of coincidence counts *R*_si_(*t*), we use


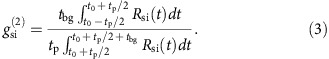


For all the cross-correlation values in this paper, we used coincidence windows *t*_p_=*t*_bg_=0.8 ns.

## Additional information

**How to cite this article:** Saglamyurek, E. *et al.* A multiplexed light-matter interface for fibre-based quantum networks. *Nat. Commun.* 7:11202 doi: 10.1038/ncomms11202 (2016).

## Supplementary Material

Supplementary Information Supplementary Figures 1-3, Supplementary Notes 1-3 and Supplementary References.

## Figures and Tables

**Table 1:** Experimental cross-correlation values.

**Mode**	**Storage**	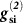 **for spectro-temporal modes**		
**#**	**Time (ns)**	**9 GHz**	**4.5 GHz**	**3 GHz**
*f*_1_	3	16.8±0.8	8.0±0.6	5.7±0.6
*f*_2_	5	16.4±0.8	7.2±0.6	5.3±0.6
*f*_3_	7	—	6.3±0.5	5.4±0.6
*f*_4_	9	—	5.2±0.5	5.0±0.6
*f*_5_	11	—	—	3.4±0.5
*f*_6_	13	—	—	3.4±0.5

Measured values of the cross-correlation function of each recalled spectral mode *f*_*i*_ for different AFC bandwidths and total numbers of modes. The storage times are given in column 2.
