# Supplementary material for: A multiplexed light-matter interface for fibre-based quantum networks
Source: Nat Commun. 2016 Apr 5;7:11202. doi: 10.1038/ncomms11202 (PMC4822043; doi:10.1038/ncomms11202)
Supplement: Supplementary Information — Supplementary Figures 1-3, Supplementary Notes 1-3 and Supplementary References. [file ncomms11202-s1.pdf]

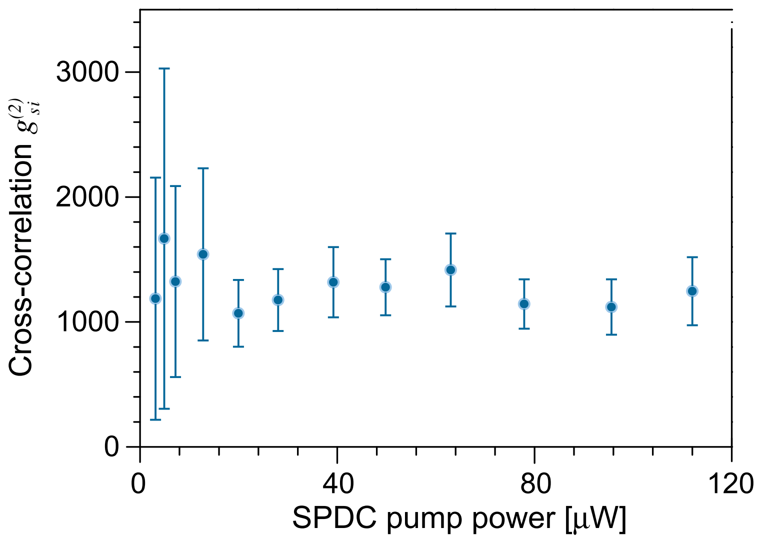

Supplementary Figure 1: Characterisation of $g_{\mathrm{si}}^{(2)}$ before storage vs. SPDC pump power. We measure the second-order cross-correlation function given in Eq. (2) as a function of SPDC pump power before storage in the AFC memory. Error bars represent the standard deviation computed from the counting statistics of the coincidence and single counts, which are assumed to obey a Poissonian distribution. All measured values of $g_{\mathrm{si}}^{(2)}$ exceed 1000, which is substantially above the classical limit of 2 for thermal light fields – having a photon number distribution following Bose-Einstein statistics). The large uncertainties of $g_{\mathrm{si}}^{(2)}$ for low pump powers are due to small photon pair generation rates in conjunction with the relatively short data accumulating times.

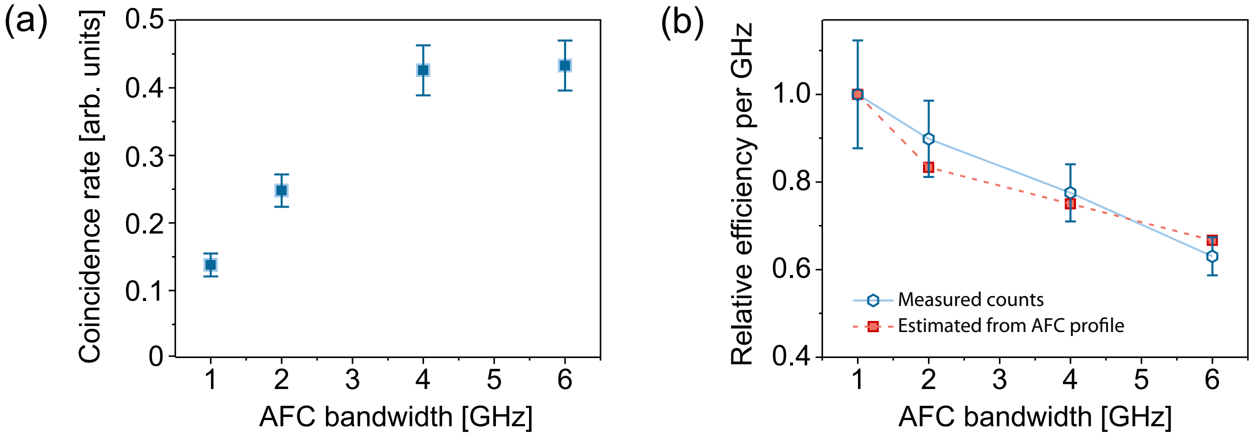

Supplementary Figure 2: Storage efficiency vs. AFC bandwidth. Due to the limited splitting and significant broadening of the Zeeman-levels – employed as shelving levels for the population that is optically pumped to tailor an AFC – the bandwidth of each individual AFC is limited. More precisely, as the AFC bandwidth increases, population removed from one edge of the AFC starts filling the troughs at its opposite edge, leading to a reduction of the recall efficiency. This behaviour can be seen from a), where the rate of detected heralded photons at the memory output is plotted as a function of the bandwidth of a single AFC at 1532 nm. As the bandwidth is increased the memory efficiency initially grows, but then reaches a plateaus. In b) we plot measured and estimated efficiencies per AFC bandwidth, normalized to 1 for a 1 GHz broad AFC. As expected, we find a monotonous decrease as the total bandwidth increases. All error bars represent the standard deviation computed from the counting statistics of the coincidence counts, which are assumed to obey a Poissonian distribution.

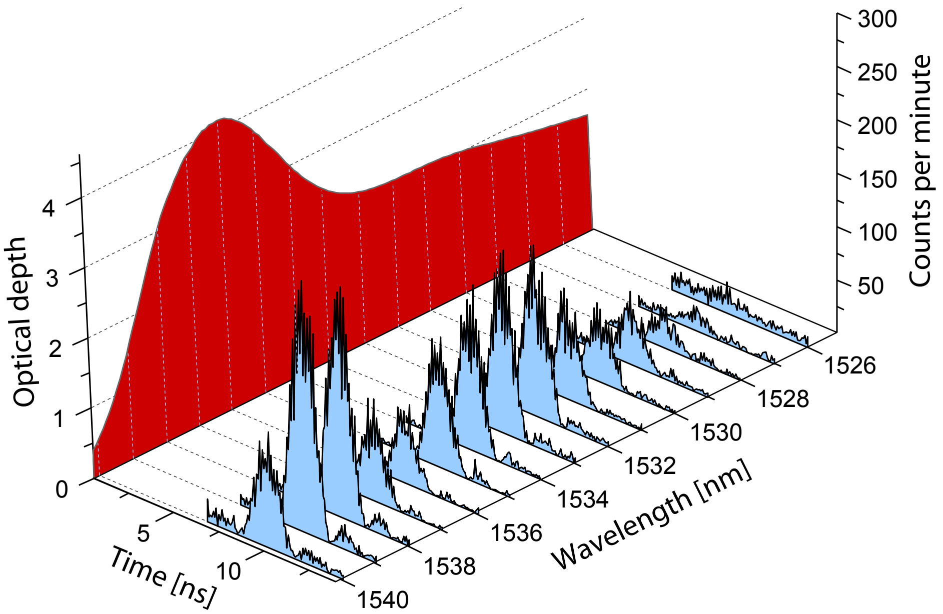

Supplementary Figure 3: Total accessible AFC bandwidth. To assess the total width of the inhomogeneously broadened absorption line of the erbium-doped fibre that is accessible for quantum storage, we tune the wavelength of the optical pumping laser to generate 1 GHz wide AFCs with 10 ns storage time over a spectral interval ranging from 1526 nm to 1540 nm wavelength. We then store 2 ns long attenuated laser pulses with mean photon number of 0.5 ± 0.1, generated at an effective mean rate of 100 kHz, in the AFC. We observe recalled photons in all cases, showing that the entire bandwidth of 14 nm is suitable for optical quantum memory (i.e. spin level lifetimes as well as optical coherence times are sufficiently large). The observed variation in the echo intensity is predominantly due to the different initial optical depths of the transition. The temperature of the fiber was 0.8 K and the magnetic field was 600 G.

SUPPLEMENTARY NOTE 1: INTEGRATED PHOTON PAIR SOURCE

In the following, we detail the structural properties and fabrication of our non-classical light source. Our heralded single photon source (HSPS) operates by creating photon pairs through spontaneous parametric down-conversion (SPDC) in a fibre-pigtailed PPLN crystal waveguide with type-0 phase matching. Although the time at which photon pairs are generated by illumination with a CW pump laser is random and cannot be known precisely, the temporal correlations between a pair of photons created by SPDC are well-defined, and the detection of one photon can be exploited to successfully herald the arrival of its twin. In our HSPS, an optical fibre couples the pump photons that are generated from a CW laser into an optical waveguide, which is located inside the lithium niobate crystal. The optical waveguide extends across the entire length of the 50 mm long, 0.5-mm thick Z-cut lithium niobate chip. In the central region of the lithium niobate chip, the ferroelectric polarization of the lithium niobate is periodically inverted over a total length of approximately 40 mm. The input pump photons are converted to photon pairs in this periodically poled region through the interaction of the pump field with this second order ($\chi^{(2)}$) nonlinear medium. In order to generate photon pairs with high efficiency through SPDC, the periodically poled region and optical waveguides were designed to satisfy the following quasi-phase matching [1,2] condition:

$$\frac{n_p}{\lambda_p} - \frac{n_s}{\lambda_s} - \frac{n_i}{\lambda_i} = \frac{1}{\Lambda} \tag{1}$$

where Λ is the period of the poled domains and n_p (λ_p), n_s (λ_s), and n_i (λ_i) are the effective refractive indices of the waveguide at the pump, signal, and idler wavelengths λ_p, λ_s, and λ_i, respectively. The effective indices of refraction of the optical waveguides at the pump, signal, and idler wavelengths were determined by performing numerical analysis. For generation of frequency degenerate photon pairs with $\lambda_{s(i)} \approx 1532$ nm from a pump photon with $\lambda_p \approx 766$ nm, the optimal period of the poled domains was observed to be approximately 17 μm.

The PPLN waveguides were fabricated using a Ti-indiffusion process, where a titanium layer was applied to the Z surface of a lithium niobate crystal and Ti-indiffusion was achieved by heating the crystal in a furnace at $T \sim 1000°$C. The PPLN waveguide width was 7 μm, and single mode operation was observed at $\lambda \sim 1532$ nm for PPLN waveguide widths < 8 μm. The photon pairs generated inside the PPLN waveguide are collected in an optical fibre, and SMF-28 fibre was used to ensure single mode propagation. In order to achieve stable and reliable optical coupling, SMF-28 optical fibre connectors were attached to the PPLN crystal using UV-cured epoxy. In order to reduce the optical coupling loss between the PPLN waveguide and the optical fibre and, therefore, increase the heralding efficiency of our single photon source, tapered and periodically segmented waveguide (PSW) geometries[3] were integrated into the optical waveguide design. A detailed description of the optical modeling that supported the development of the PPLN waveguide and the PSW taper designs used in our HSPS has been presented in a prior publication, along with supporting optical test and characterization data[4].

For this work, the CW pump power level was limited to less than ~ 100 μW. This power level constraint was a consequence of the photorefractive effect. For pump power levels < 100 μW, the

rate at which photon pairs are generated scales linearly with pump power. At pump power levels $> 100\ \mu$W the rate of photon pair generation no longer scales linearly with pump power, and the pair generation rate will start to saturate and eventually degrade as the power level is increased. To enable operation at higher power levels, our team is currently designing Ti-indiffused PPLN waveguides to operate at $T > 120°$C, because photorefractive effects are significantly reduced at elevated temperatures. We are also designing, fabricating, and characterizing proton-exchanged and Zn-indiffused PPLN waveguides, which are less sensitive to photorefractive effects than Ti-indiffused waveguides.

SUPPLEMENTARY NOTE 2: EFFICIENCY, STORAGE TIME AND BANDWIDTH OF THE AFC MEMORY

The efficiency of our current AFC memory implementation, which is based on the re-emission of the stored photon in forward direction, is theoretically limited to 54%. One method to overcome this limit is to incorporate the AFC into an impedance-matched cavity and retrieve the photon "backwards", in which case the memory efficiency may reach 100%[5]. From a practical viewpoint the efficiency is furthermore limited by the imperfect preparation of the AFC, which leads to irreversible photon absorption, as described in the Methods. There are three main factors contributing to this absorption.

The first arises from the insufficient lifetime of the Zeeman sublevels (~ 1.3 s) compared to that of the excited level (11 ms). More precisely, by the end of the 300 ms wait time which is set to avoid spontaneously emitted photons, i.e. noise, due to decaying atoms a significant portion of the Zeeman level population has decayed, filling the AFC troughs and hence absorb photons in a non-reversible manner. As we detail in the Supplementary Information of [6], for typical settings in our experiments, the contribution to the overall background coming from insufficient Zeeman level lifetime is about 65%. However, as we reported in [7], it is possible to obtain longer lifetimes for the Zeeman sublevels, and thus almost completely eliminate this contribution background, by lowering the temperature and lowering the erbium concentration.

A second factor is the small branching ratio, i.e. the probability of an atom during spectral hole burning to decay into the spin state (Zeeman sublevel) that differs from that before excitation. This results in incomplete population transfer among the sublevels, and hence residual absorption in the AFC background. With an increased Zeeman level lifetime at lower temperatures, there would be more time available for optical pumping and this contribution to the background could also be reduced.

The third factor contributing to the residual absorption is the restriction imposed by the GHz-bandwidths of the AFCs compared to the small splitting of the Zeeman sublevels (for typical magnetic fields of 600 G) in combination with the very large variation of the splitting (i.e. broadening of the Zeeman splitting) due to disorder in the glassy host. This effect leads to cross pumping

during the AFC preparation i.e. a considerable overlap of the AFC troughs (spectral sections with decreased absorption) with the very wide antiholes (sections with increased absorption). Consequently, a significant number of the atoms cannot be removed from the spectral interval of the AFC. As we present in Supplementary Figure 2 for a fixed magnetic field, the impact of this factor on the AFC efficiency decreases as the bandwidth is reduced from 6 GHz to 1 GHz. This observation highlights that a larger ratio of magnetic field to bandwidth would allow a significant improvement of the efficiency. For instance, as we showed in [7], the reduction of the bandwidth while keeping the magnetic field at 600 G does indeed lead to much less cross pumping. However, this approach obviously also reduces the time-bandwidth product unless we simultaneously increase the storage time, which is limited by the optical coherence time. (The latter can be improved by lowering the temperature.) Conversely, focussing instead on increasing the level splitting by means of increasing the strength of the applied magnetic field, our studies in [7] show that for an AFC with a constant bandwidth of about 1 GHz, the cross-pumping can also be decreased. Moreover, as we report in a forthcoming manuscript[8], the effective coherence time does not exhibit strong magnetic field dependence. The field increase does, however, result in a reduction of the persistent hole lifetime[6]. This is caused by the stronger coupling of low-frequency two-level system (TLS) modes to erbium spins with the larger Zeeman splitting, which, once more, points to the temperature as a critical factor. Lowering the temperature would diminish these interactions and make it possible to achieve long persistent hole life-times at large magnetic fields as well.

SUPPLEMENTARY NOTE 3: QUANTUM CORRELATIONS AND STORAGE IN AFC MEMORY

Below we discuss how the parameters that govern the storage of photons generated by the SPDC source determine the measured cross correlations both before and after storage. We start by restating Eq. (2) in the main text for $g^{(2)}_{\mathrm{si}}$ in terms of experimentally measurable quantities

$$g^{(2)}_{\mathrm{si}} = \frac{R_{\mathrm{si}}}{R_{acc}} \;, \tag{2}$$

where R_{si} is the photon pair coincidence count rate and R_{acc} is the accidental count rate, which is identical to the quantity $R_{\mathrm{si}}(t \neq 0)$ defined in the Methods section of the main text. R_{acc} has in principle contributions from multi pair emissions of the source as well as detector dark counts. However, at the rate of 10 Hz, the latter is negligible. For the case in which the fibre memory is bypassed, R_{si} can be decomposed as

$$R_{\mathrm{si}} = 0.5\eta_c^2\eta_d^2 R + R_{acc} \;, \tag{3}$$

where η_c, η_d and R are the collection efficiency of signal and idler photon, the detection efficiency of the SNSPDs, and the photon pair generation rate, respectively. We extract η_c from measurements of the coincidence and signal count rates, R_{si} and R_s, respectively, and the detection efficiency of the SNSPD on the *idler* side to be about 8.6%. As shown in Fig. 1 we measure $g^{(2)}_{\mathrm{si}}$ values in excess of 1000, which points to R_{acc} being only a very small fraction of R_{si}.

With the addition of the AFC memory for the storage of one member of the photon pair, this expression can be written as

$$R'_{\mathrm{si}} = 0.5\eta_c^2\eta_d^2\eta_s R + R'_{acc}\ , \tag{4}$$

where η_s is the system efficiency for the recalled photon in a specific spectral and temporal mode, and R'_{acc} is the accidental coincidence rate measured when the AFC memory is operated. By inspecting Eqs. 2 - 4, we find that $g_{\mathrm{si}}^{(2)}$ will be reduced if $R'_{acc} > \eta_s R_{acc}$. Hence, $g_{\mathrm{si}}^{(2)}$ will decrease either due to a low η_s or due to additional noise induced by the memory. In the following we elaborate on both effects.

First, the system efficiency η_s can be considered a product of three factors: the retrieval efficiency of the AFC memory η_m, the transmission factor η_t that takes into account the optical loss in the erbium-doped fibre due to splices and bending as well as in the optical circulator, and a filtering factor f ($0 \leq f \leq 1$), which is given by ratio of the bandwidth of the AFC section of interest and the bandwidth of the input photons (50 GHz). While η_t, which we measured to be 14%, is a constant in all experiments, both η_m and f depend on the preparation of the AFC. It is obvious that f is directly proportional to the AFC bandwidth, and we also find that the recall efficiency η_m decreases with increasing bandwidth of a single AFC, as described in Supplementary Fig. 2. Of course, a reduction of η_s does not necessarily decrease $g_{\mathrm{si}}^{(2)}$ unless a noise source that is independent of η_s is present, e.g., ultimately, detector dark counts.

Provided the transmission loss and the filtering factor are known, the AFC retrieval efficiency can directly be determined for any spectro-temporal mode by time-resolved measurements of R_{si} and R'_{si} (corresponding to the cases with and without memory, respectively), since η_m is given by

$$\eta_m = \frac{1}{\eta_t f}\frac{R'_{\mathrm{si}} - R'_{acc}}{R_{\mathrm{si}} - R_{acc}}. \tag{5}$$

This formula, combined with independently determined values for the filtering factor and η_t, is used to compute the memory efficiency η_m plotted in Fig. 4 in the main text and Supplementary Fig. 2.

Next, we investigate the noise sources contributing to R'_{acc}. We start be writing the accidental coincidence rate as

$$R'_{acc} = \eta_s R_{acc} + R_{noise}\ , \tag{6}$$

where R_{acc} is the same as that defined in Eq. (3) — note that it appears as a product with the system efficiency η_s. Hence, the cross-correlation function cannot be reduced due to any source of accidental coincidences that is already present without the memory. However, the term R_{noise}, which includes two sources that are unique to the operation of the memory, leads to a reduction of $g_{\mathrm{si}}^{(2)}$. i) The first source of noise is the light spontaneously emitted from the small fraction of atoms that are excited during optical pumping and remain in the excited state after 300 ms of waiting time. In principle, this noise and the resulting accidental coincidences can be further suppressed by extending the waiting time and better optimization of the intensity of the optical pumping light. ii) The second source of noise is due to SPDC photons that are absorbed by atoms that do not

contribute to the reversible mapping via the AFC i.e. atoms that either remain in a trough (and hence contribute to the AFC background), or that are outside of the AFC section. These photons are spontaneously re-emitted within the lifetime of the excited level (10 ms) and can be accidentally detected in coincidence with the heralded photons.

To highlight the effect of spontaneous-emission noise, we rewrite Eq. (2) taking into account Eqs. (4) and (6)

$$g^{(2)}_{\mathrm{si}} = \frac{R'_{\mathrm{si}}}{R'_{acc}} = \frac{0.5\eta_c^2\eta_d^2 R + R_{acc} + R_{noise}/\eta_s}{R_{acc} + R_{noise}/\eta_s}. \tag{7}$$

This shows that even though the number of spontaneously emitted photons may be much smaller than the number of input SPDC photons within the AFC bandwidth, they can nevertheless cause an significant reduction in $g^{(2)}_{\mathrm{si}}$. This is mainly a consequence of our small current system efficiency η_s, on the order of 0.1%.

There is a number of ways to reduce the effect of spontaneous emission noise, one obviously being to increase the AFC efficiency. Another path is to reduce the bandwidth mismatch between the input photons and the tailored AFC sections. In spectral multiplexing applications this could be accomplished by using multimode sources that produce photons only in spectral modes that are matched to the AFC sections. Another solution is to implement additional filtering to remove the spectrally unmatched portions of the input photons prior to the memory.

SUPPLEMENTARY REFERENCES

1. Lim, E., Fejer, M., & Byer, R. Efficient quasi-phase-matched blue second-harmonic generation in $LiNbO_3$ channel waveguides by a second-order grating. *Electron. Lett.* **25** 174 (1989).

2. Webjorn, J., Laurell, F., & Arvidsson, G. Domain inversion in MgO-diffused $LiNbO_3$. *J. Lightwave Technol.* **7** 1597 (1989).

3. Castaldini, D., Bassi, P., Tascu, S., Aschieri, P., De Micheli, M.P., & Baldi, P. Soft-Proton-Exchange Tapers for Low Insertion-Loss $LiNbO_3$ Devices. *J. Lightwave Technol.* **25** 15881593 (2007).

4. Oesterling, L., Monteiro, F., Krupa, S., Nippa, D., Wolterman, R., Hayford, D., Stinaff, E., Sanguinetti, B., Zbinden, H., & Thew, R. Development of a Photon Pair Source using Periodically Poled Lithium Niobate and Fiber Optic Components. *Journal of Modern Optics* **62** 1-10 (2015)

5. Sabooni, M., Li, Q., Kröll, S. & Rippe, L. Efficient Quantum Memory Using a Weakly Absorbing Sample. *Phys. Rev. Lett.* **110** 133604 (2013).

6. Saglamyurek, E. *et al.* Quantum storage of entangled telecom-wavelength photons in an erbium-doped optical fibre. *Nat. Photon.* **9**, 83–87 (2015).

7. Saglamyurek, E. *et al.* Efficient and long-lived zeeman-sublevel atomic population storage in an erbium-doped glass fiber. *Phys. Rev. B* **92**, 241111 (2015).

8. Viessier, L., Falamarzi, M., Lutz, T., Saglamyurek, E., Thiel, C. W., Cone, R. L., Oblak, D., & Tittel, W. Optical decoherence and spectral diffusion in an erbium-doped silica glass fiber featuring long-lived spin sublevels. *in preparation* (2016).
